# Using the Ages & Stages Questionnaire to assess later effects of an infant intervention promoting language in primary care

**DOI:** 10.1186/s12887-023-03953-y

**Published:** 2023-04-06

**Authors:** Gretchen J. Domek, Lori Silveira, Helene Kuffel, Lauren Heller Szafran, Andrea Jimenez-Zambrano, Bonnie W. Camp

**Affiliations:** 1 Department of Pediatrics, University of Colorado Anschutz Medical Campus, 13123 E 16th Ave, B065, Aurora, CO 80045 USA; 2 Center for Global Health, Colorado School of Public Health, Mail Stop A090, 13199 E Montview Blvd, Suite 310, Aurora, CO 80045 USA; 3 University of Colorado School of Medicine, 13001 E 17th Pl, Aurora, CO 80045 USA; 4 Adult & Child Center for Outcomes Research & Delivery Science, University of Colorado Anschutz Medical Campus, 1890 N Revere Ct, F443, Aurora, CO 80045 USA; 5 Department of Psychiatry, University of Colorado Anschutz Medical Campus, 13001 E 17th Place, Aurora, CO 80045 USA

**Keywords:** Infant development, Language development, Social-emotional development, Parent-child interaction, Primary health care

## Abstract

**Background:**

Positive relational experiences during infancy have a profound impact on child development and are critical for future health and school readiness. We have been evaluating a simple finger puppet intervention that takes one minute and costs $1USD to deliver in the primary care setting to promote caregiver-infant interactions. We explored using developmental trajectories to determine later outcomes of our early intervention program by comparing trajectories to age 36 months to assess optimal intervention timing when delivered in early versus late infancy.

**Methods:**

Three cohorts were enrolled and given a puppet at 2 months (early intervention) and 6 or 12 months (late intervention). Child development was assessed using the Ages & Stages Questionnaires (ASQ-3), which were independently collected during well visits. Scanned ASQ-3 forms from 2 to 36 months were obtained retrospectively through the electronic medical record. To compare longitudinal scores at different ages, all raw scores were first converted to z-scores. Longitudinal mixed effects models examined the trajectories of participant ASQ-3 scores over time by comparing the average intercepts and slopes.

**Results:**

Of 180 children enrolled, 172 (96%) completed 2 or more ASQ-3 questionnaires and were included in the analysis, with a mean of 4.9 and a total of 843 questionnaires. Most children (85%) were on government-sponsored insurance. There were no statistical differences comparing cohort intercepts, while early intervention had a significant difference in slope compared to late intervention for the Personal-Social domain (0.12, *p*=0.018), resulting in higher predicted scores at 36 months. Early compared to late intervention had a difference in slope approaching significance for Communication (0.14, *p*=0.056) and the combined non-motor score (0.33, *p*=0.052). There were no significant differences in slope for Problem Solving (0.05, *p*=0.48), Gross Motor (-0.009, *p*=0.84), Fine Motor (0.06, *p*=0.22), and total ASQ-3 (0.32, *p*=0.17) scores.

**Conclusions:**

Finger puppets may provide a simple and scalable way to encourage responsive caregiver-infant interactions promoting language and social-emotional development, especially when provided in early versus late infancy. Our trajectory analysis also demonstrates a useful and potentially cost-effective approach to evaluating long-term developmental outcomes of an early intervention.

## Introduction

Positive relational experiences during infancy have a profound impact on early brain and child development and are critical for future health, school readiness, and academic success. Infant and parent brains are biologically wired to connect socially and emotionally from the very beginning. Foundational aspects of brain circuitry and architecture form during early infancy and are critically shaped by experiences with positive social interactions and attentive caregiving [1]. Furthermore, neural networks in adults are reorganized through human parenting [2], contributing to a process termed ‘bio-behavioral synchrony’ or the co-wiring of parent and infant brains into a more synchronous entity [3]. This includes maternal behaviors promoting early social and communicative development, such as gaze and ‘motherese’ vocalizations, that are genetically and hormonally primed from birth. These neural connections are then formed and modified by environmental interactions, with adverse environments that interfere with the initial parent-infant relationship being detrimental to future development.

The recent and revised American Academy of Pediatrics (AAP) policy statement on childhood toxic stress endorses a paradigm shift toward a relational health framework, promoting safe, stable, and nurturing relationships to buffer adversity and build resilience [4]. This policy statement highlights the important role of the pediatric community in prioritizing relational health as an integral component of pediatric care, research, and advocacy to “proactively build healthy, resilient children” [4]. Pediatric primary care has been increasingly recognized as a wide-reaching venue for fostering healthy relationships that promote social-emotional well-being and school readiness [5, 6]. A recent meta-analysis of programs teaching adults how to be more responsive toward their children showed that parental responsivity can be taught by researchers in a scalable way through short, focused programs that do not require more expensive and involved methods, such as longer-term home visiting programs [7]. This review found that the most effective programs involved parents learning about and observing responsive caregiving and then encouraging further home practice of these responsive parent-child interactions [7]. Such short, focused programs could potentially be implemented in a scalable way in the primary care setting. Important examples of universal primary preventions supporting the positive parenting approach to relational health in pediatrics include programs such as Reach Out and Read (ROR) [8], the Video Interaction Project (VIP) [9–11], and HealthySteps [12], which have all been shown to enhance social-emotional development [9, 11, 13, 14].

Our study team has been evaluating a very simple, low-cost finger puppet intervention that takes less than one minute and costs $1USD to deliver in the primary care setting to promote language-rich caregiver-infant interactions [15–19]. We first developed the intervention as part of a larger early childhood program in a low-resource, rural Guatemalan population where it was received with high satisfaction [15]. We then conducted two pilot studies in a primarily low-income population of U.S. families where we introduced puppets during infant well child visits. Our initial results have been encouraging, suggesting that intervention families may have better outcomes related to maternal depression, the cognitive home environment, and early language development during the first year of life, especially for families who reported using the puppet more [16–19]﻿. We hypothesize that early puppet usage will build a critical foundation for future language, cognitive, and social-emotional development by increasing both the quantity and quality of infant-directed speech (e.g., exaggerated sounds and facial expressions, a positive affect, and social interchanges), allowing and encouraging caregivers to say silly and repetitive vocalizations. When caregivers experience a positive infant reaction to these vocalizations, we hypothesize that they will become more likely to continue responsive social interactions, with or without the puppet. This could exponentially increase the intervention’s strength by fostering the critical development of neural networks in both infants and parents who are biologically primed for these early interactions. The puppet is simply a means to introduce the importance of talking and facilitate early caregiver-infant interactions, while the real intervention is the caregiver’s voice and subsequent talking that takes place long after the puppet has been provided. In this pilot study, we explored the use of developmental trajectories in determining the later effectiveness of our early intervention program. We hypothesized that earlier delivery of the intervention would have better long-term developmental outcomes. We explored associations between providing puppets in the first compared to second half of infancy with developmental trajectories between 2 to 36 months of age to better understand optimal timing of the intervention.

## Methods

### Study design

This research was a secondary analysis for a pilot study that used an experimentally staged introduction, similar to a delayed intervention or stepped wedge design [20], where the intervention was introduced in a staggered manner at different time points to all participants. Study procedures were approved by the Colorado Multiple Institutional Review Board (Protocol 18-0792). Written informed consent was obtained from all caregivers prior to participation.

### Study participants

Three convenience samples were recruited during routine infant well visits at a university-affiliated primary care clinic that serves a primarily low-income population. The early intervention cohort was enrolled at the 2-month well visit between May to August 2018. Two additional cohorts serving as late intervention for this analysis were enrolled at the 6-month well visit between August 2018 to February 2019 and the 12-month well visit between February to August 2019. Families were only approached for enrollment once during the study period. We recruited 70 participants at 2 months, 60 participants at 6 months, and 50 participants at 12 months to reach our participation goals as part of the main pilot study. Eligible infants were born full-term (≥37 weeks), weighed at least 2500 grams at birth, and had no chronic condition or exposure known to affect neurodevelopment (e.g., neonatal asphyxia, major congenital malformation, or in utero exposure to drugs or alcohol), which were confirmed by chart review. Caregivers were at least 18 years old, fluent in English or Spanish (but these did not need to be the primary language), and expected to stay at the same clinic for the child’s first year of life during the main study period. Sociodemographic risk was assessed using a cumulative risk index previously developed by a member of the study team [21]. The cumulative risk index combines multiple social determinants into a single composite variable scoring one point for each of the following factors collected at enrollment: 1) single, divorced, or separated marital status, 2) Hispanic or non-white maternal race/ethnicity, 3) maternal education less than high school/GED, 4) government-sponsored health insurance. Systemic barriers may place children from racial and ethnic groups at increased risk for poor development, which is why this factor was included. Cumulative risk scores range from 0-4 and are classified as low-risk (0-2) or high-risk (3-4) for child development problems [21].

### Procedures

Research assistants approached families while waiting for their infant well visit. After eligibility was confirmed and written informed consent obtained, families completed a brief sociodemographic questionnaire. Each cohort was introduced to the intervention at the end of their enrollment visit. Participants were contacted by phone two weeks after enrollment to complete a caregiver feedback survey, with up to five contact attempts made. Parenting and child development primary outcome measures were assessed during the 6- and 12-month visits as part of the main study and are presented elsewhere [18, 19]. No incentive was provided at the shorter 2-month visit. Small cash reimbursements ($20) for time were provided to all participants after the lengthier 6- and 12-month visits. Study data were managed using REDCap (Research Electronic Data Capture), a secure and web-based electronic data capture tool [22]. All participants received the same standard care delivered by clinic providers who were blinded to study cohort. The clinic provides Reach Out and Read [8] to all children starting at the 6-month well visit and the HealthySteps [12] program to some patients generally starting by 6 months of age. Study cohorts had equal opportunities to participate in these programs, with 4 (6%) early intervention and 12 (12%) late intervention participants enrolled in HealthySteps.

### Finger puppet intervention

All participants received an animal finger puppet valued at $1USD at their enrollment visit. Research assistants followed a short script explaining that it was important to talk to infants so that children learn to talk and become ready for school. They explained that the puppet could help caregivers and other family members talk and should be used as often as possible. Research assistants did not model the vocalizations or ask parents to practice during the visit. Participants were provided a one-page list of suggested puppet activities (e.g., make silly sounds, play peek-a-boo, walk around the house and explore, look at pictures in books and magazines, gentle touch and massage, sing songs and say rhymes, explore the outdoors, tummy time, and read simple books) in English or Spanish to take home and use if desired. This initial instruction took less than one minute. Intervention dosage was established during the caregiver feedback survey based on the following question and answers: “On average, how often have you used your puppet in the past 2 weeks: several times a day, once a day, a few days a week, or once a week or less?" Dosage was classified as high for families using the puppet at least daily, low for families using the puppet less than daily, and unknown for families not completing the survey.

### Outcome measures

Child development was assessed using the Ages & Stages Questionnaires, Third Edition (ASQ-3) [23]. The ASQ-3 is the most widely used parent-report developmental screening tool that can be used with children ages 1 to 66 months, with 21 possible versions depending on the child’s age [24]. It is available in several languages, including English and Spanish, and has demonstrated good psychometric properties [23, 25, 26]. Each questionnaire takes around 10-15 minutes for parents to complete and assesses five developmental domains: Communication, Gross Motor, Fine Motor, Problem Solving, and Personal-Social. Each domain contains six items that are marked “yes” (10 points), “sometimes” (5 points), or “not yet” (0 points) based on whether the child is performing the described skill, with a possible raw score of 60 points per domain. ASQ-3 questionnaires were administered, scored, and entered into the medical record independently by blinded clinic staff and providers as part of the clinic’s routine developmental screening done at all well child visits between 2 months to 5 years. For this study, scanned copies of paper ASQ-3 forms were obtained retrospectively through the electronic medical record. Data were rescored by a member of the research team and entered into REDCap, with visual checking done by a second researcher to ensure accuracy. We followed the ASQ-3 manual’s recommended score adjustment when up to two item responses in a domain were omitted by replacing missing answers with the mean of the answered questions in the same domain [23].

### Data analysis

Sociodemographic characteristics were summarized using means (standard deviations) for continuous variables and counts (percents) for categorical variables. We did not calculate sample size power calculations for this secondary analysis or correct for multiple comparisons as this was a pilot study to establish estimates for effectiveness. Children with two or more completed ASQ-3 questionnaires were included in the analysis assessing longitudinal associations between domain scores and child age for the intervention. We first assessed the three cohorts separately but did not have the power to detect significant differences. As an exploratory analysis, we combined the two late intervention cohorts enrolled at 6 or 12 months because we hypothesized that earlier delivery would have greater impact. We also explored developmental trajectories for high- versus low-dosage participants for each cohort. We included ASQ-3 results from the highest attended well visits between 2 to 36 months (which included 2, 4, 6, 12, 18, 24, and 36 months). We analyzed each domain score (60 points possible), a combined score for the three non-motor domains (Communication, Problem Solving, and Personal-Social) (180 points possible), and a total score for all domains (360 points possible). To compare longitudinal ASQ-3 scores for children of different ages, all raw scores were first converted to z-scores, which is the difference between the child’s observed value and the study population’s mean value divided by the standard deviation value of the study population. Longitudinal mixed effects models, which are an extension of classic regression analysis but allow for the analysis of correlated data and differing follow-up times, were developed to examine the trajectories of the participant ASQ-3 scores over time between the early intervention (enrolled at 2 months) and late intervention (enrolled at 6 or 12 months) cohorts as well as the high-dosage and low-dosage cohorts. Each participant was allowed to have their own slope and intercept, and the average intercepts and slopes were compared. Interaction terms were included in the model with age and the intervention cohort or age with cumulative risk score. Contrast statements were constructed to compare slopes and intercepts between cohorts. A two-tailed *p*-value <0.05 was considered statistically significant. Analyses were conducted using SAS (SAS 9.4, SAS Institute, Cary, NC).

## Results

### Participant characteristics

**Fig. 1:**
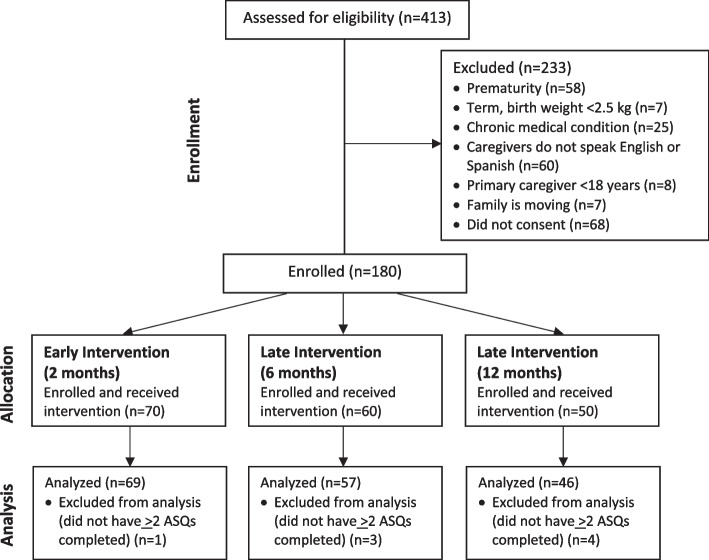
Study Diagram

A total of 413 children were screened for eligibility, and 248 (60%) were eligible to participate. (Figure 1) For early intervention, 76 families were approached and 70 (92%) enrolled at 2 months; 69 (99%) completed two or more ASQ-3 questionnaires. For late intervention, 172 families were approached and 110 (64%) enrolled at 6 or 12 months; 103 (94%) completed two or more ASQ-3 questionnaires. Table 1 presents sociodemographic characteristics for participants included in this analysis. Most mothers considered themselves Hispanic/Latino (*n*=63, 37%) or Black/African American (*n*=59, 34%). Most children were on government-sponsored insurance (*n*=147, 85%). There were no significant sociodemographic differences between study cohorts, and therefore, these individual variables were not tested as moderators. Using the cumulative risk index, 112 (65%) families scored low-risk (CR 0-2) and 60 (35%) high-risk (CR 3-4) for developmental delays. There were no differences in ASQ-3 scores and trajectories between families scoring high- versus low-risk on the cumulative risk index. Seventy-two percent (*n*=123) of families completed the caregiver feedback phone survey. Half of families (*n*=62, 50%) reported using the puppet daily and were considered high dosage. Given the small sample size in each cohort, we were not adequately powered to detect differences associated with dosage and intervention timing.

**Table 1:** Sociodemographic Characteristics of Study Participants

	**Early Intervention**	**Late Intervention**	***p*-value**
	(*n*=69) n (%)	(*n*=103) n (%)	
**Child Characteristics**			
Gender			
Male	33 (47.8%)	37 (35.9%)	0.15
Female	36 (52.2%)	66 (64.1%)	
**Maternal Characteristics**^a^			
Age (years), mean (SD)	26.7 (6.1)	27.8 (6.3)	0.23
Has high school diploma/GED			
Yes	60 (87.0%)	84 (81.6%)	0.40
No	9 (13.0%)	19 (18.4%)	
Marital status			
Married	35 (50.7%)	46 (44.7%)	0.57
Living with partner, not married	19 (27.5%)	29 (28.2%)	
Divorced/separated/widowed	1 (1.4%)	6 (5.8%)	
Single/never been married	14 (20.3%)	22 (21.4%)	
Ethnicity/race			
White, not of Hispanic origin	12 (17.4%)	12 (11.7%)	0.12
Hispanic/Latino	26 (37.7%)	37 (35.9%)	
Black/African American	23 (33.3%)	36 (35.0%)	
Asian	0 (0.0%)	4 (3.9%)	
American Indian/Alaska Native	1 (1.4%)	3 (2.9%)	
Native Hawaiian/Pacific Islander	2 (2.9%)	3 (2.9%)	
Multiracial	5 (7.2%)	8 (7.8%)	
**Household Characteristics**			
Number of children, mean (SD)	2.2 (1.9)	2.1 (1.9)	0.52
Number of languages spoken			
1	40 (58.0%)	52 (50.5%)	0.33
2	28 (40.6%)	45 (43.7%)	
3 or more	1 (1.4%)	6 (5.8%)	
Primary language spoken			
English	56 (81.2%)	70 (68.0%)	0.12
Spanish	9 (13.0%)	18 (17.5%)	
Other	4 (5.8%)	15 (14.6%)	
Child’s insurance			
Government-sponsored	56 (81.2%)	91 (88.3%)	0.20
Private	13 (18.8%)	12 (11.7%)	
Cumulative risk score^b^			
0 – 2 (low risk)	48 (69.6%)	64 (62.1%)	0.33
3 – 4 (high risk)	21 (30.4%)	39 (37.9%)	

^a^One primary caregiver was a grandmother. All other primary caregivers were the child’s mother

^b^Number of the following risk factors: single/divorced/separated marital status, Hispanic or non-white maternal race/ethnicity, maternal education less than high school/GED, and government-sponsored health insurance

### Primary outcomes

**Fig. 2:**
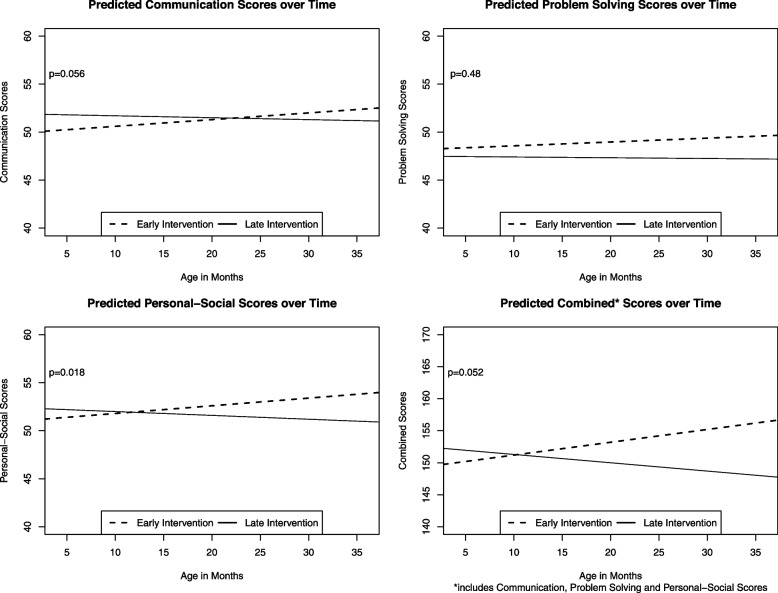
Developmental Trajectories Comparing Non-Motor ASQ-3 Domains Between Early and Late Intervention

The 172 children included in the analysis completed 843 total ASQ-3 questionnaires, with each participant completing an average of 4.9 questionnaires during the 7 possible included visits. Figure 2 shows the mixed effects longitudinal modeling between the early and late intervention cohorts and ASQ-3 domain scores for the non-motor domains (Communication, Problem Solving, Personal-Social, and a combined non-motor score for these three domains). There were no statistical differences comparing cohort intercepts, while early intervention had a significant difference in slope compared to late intervention for the Personal-Social domain (0.12 [95% CI, 0.02, 0.2], *p*=0.018). Early compared to late intervention had a difference in slope approaching significance for Communication (0.14 [95% CI, 0, 0.3], *p*=0.056) and the combined non-motor score (0.33 [95% CI, 0, 0.9], *p*=0.052). There were no significant differences in slopes for Problem Solving (0.05 [95% CI, -0.1, 0.2], *p*=0.48), Gross Motor (-0.009 [95% CI, -0.1, 0.08], *p*=0.84), Fine Motor (0.06 [95% CI, -0.04, 0.2], *p*=0.22), and total ASQ-3 (0.32 [95% CI, -0.1, 0.8], *p*=0.17) scores.

## Discussion

Our pilot study showed that a primary care-based intervention using finger puppets to promote caregiver-infant interactions was associated with improved social-emotional developmental trajectories in the first three years of life when delivered in early infancy (2 months) compared to later infancy (6 or 12 months). A central aspect of social-emotional development is language, with prelinguistic skills, vocabulary development, and social-emotional competencies being interrelated [27, 28]. Our intervention stimulates relational health highlighted by talking and the encouragement of early language. The intervention is exceptionally simple, low-cost, and scalable, with the potential for widespread dissemination in the primary care setting compared to existing interventions. While we found improved trajectories for the early compared to late intervention cohort that approached significance (*p*=0.05-0.06) for communication skills and combined communication, cognitive, and social-emotional development, only social-emotional trajectories were statistically significant. The improved trajectories may indicate positive and sustained developmental changes that progress over time when the intervention is delivered earlier. The 36-month ASQ was previously found to predict later IQ scores at ages 5 to 6 years in the general population, suggesting that our findings may have long-term implications for school-readiness [29].

Our study suggests that an earlier introduction of the intervention, as early as 2 months, was optimal. While there may still be important benefits to delivering the intervention in later infancy, we were not sufficiently powered to show a linear increase in slope as the intervention was introduced earlier. Our previous findings have suggested that families who received a puppet at 2 or 6 months scored similarly in parenting and child development outcomes at 12 months [19]. It may be that the benefits of receiving the intervention earlier increase and become more apparent over time, which we are now seeing by 3 years of age in this study. Infants as young as 2 months listen preferentially to human speech compared to acoustically similar non-speech sounds [30], with important advantages of hearing words over tones for language development already evident by 3 months [31]. Additionally, social development increases most rapidly during infancy, with dramatic gains seen between the newborn period and the third month of life when children become active participants in their social worlds (e.g., cooing, gazing, smiling, and co-vocalizing) [32]. We have hypothesized that introducing puppets as an avenue to increase parent language at 2 months of age will have the greatest impact by taking advantage of this early social period in infant development when the human auditory system already prefers speech and prior to the emergence of advanced motor skills during the later half of the first year when children become more focused on grasping and manipulating objects during play [33]. While this study suggests this may be the case, longitudinal studies will be important to determine long-term impacts and to explore whether some families might also benefit from receiving the intervention in later infancy.

Our longitudinal mixed effects model demonstrates an important approach to evaluating potential long-term outcomes of an early intervention. This trajectory analysis was similar to other recent child development studies. Lamsal et al. (2018) [34] assessed longitudinal associations between ASQ domain scores and child age using fixed effects regression modeling, similarly presenting predicted ASQ domain scores for children over time. Cates et al. (2018) [10] compared trajectories of parent cognitive stimulation over time as measured by different versions of the StimQ [35] using multilevel modeling with calculated z-scores for StimQ subscale and total scores. Evaluating longitudinal relationships throughout early development, such as these studies have done, can provide a more comprehensive assessment during this critical developmental period rather than looking at outcomes at only one point in time. Furthermore, any repeated developmental screening tool used in primary care could be used in a trajectory analysis as a potentially cost-effective outcome measure, especially for an early intervention where effects on development are not expected to be readily seen until the child is older. Our study was strengthened by the high follow-up rates for primary care visits and ASQ-3 completions, with each child completing an average of nearly five ASQ-3 questionnaires at different time points during the first three years adding to a more robust longitudinal assessment.

Our studies have determined dosage through parental reports of puppet usage two weeks after receiving the intervention because we hypothesize that early usage is more likely to lead to an increase in future parent-infant interactions. For example, if caregivers use the puppet shortly after receiving it and experience a positive infant reaction to their voice, they could exponentially increase the intervention’s strength by continuing these language-rich interactions even without the puppet. While our previous study results suggest that high- dosage participants demonstrated superior outcomes related to child development [16, 18, 19], we did not see similar effects in this analysis which had more outcome scores collected by blinded providers. It is possible that a social desirability bias is partly responsible for this dose-response relationship seen in our other studies. Future studies should explore the best way to determine intervention dosage (such as usage logs, parent-child interaction scales, or a computerized automated analysis to quantitatively measure parent-child interactive talk after receiving the intervention) and whether higher dosage positively impacts outcomes.

The ASQ-3 is a developmental screening tool based on parental report. Traditional methods for developmental assessments include face-to-face standardized evaluations with highly trained examiners that are costly, labor-intensive, and time-consuming. Parent report screening tools, on the other hand, can be cost-efficient, reliable, and valid measures for evaluating early development. Parents are often valuable informants as they are generally the first to notice emerging developmental skills in their child. Shah et al. (2016) [36] recently found that most studies of U.S. primary care-based interventions promoting positive parenting behaviors used self-report parenting measures. The ASQ-3 has been used extensively in clinical and research settings with demonstrated good psychometric properties, including in the general pediatric population like our study [23, 25, 26]. The psychometric properties have also been shown to improve when used in older children including the 18-, 24-, and 36-month questionnaires that we assessed [25, 26]. An additional strength of our secondary analysis was that the ASQ-3 results were collected independently by blinded clinic providers as part of routine well care, eliminating a potential source of bias.

Our study had important limitations. Our smaller sample size prevented us from being able to analyze the three study cohorts separately. In this analysis, we combined the two late intervention cohorts because we hypothesized that earlier delivery in the first half of infancy would have better developmental outcomes. Future studies with a larger sample size could examine this potentially important linear relationship. Although this was a secondary analysis, assessing these data longitudinally allows us to get estimates at each timepoint to power future studies. While the intervention appeared to have the greatest impact when delivered in early infancy, we do not know if there were also benefits when delivered later in the first year. Furthermore, although our predicted improvements in developmental screening scores were clinically small at 36 months, the three study cohorts all received a puppet by 12 months of age and a greater effect might be found if comparing our early intervention participants to a control group not receiving the intervention. Our study design had logistical, practical, and financial advantages of phased enrollment and follow-up, but a selection bias may have occurred as recruitment was staggered and children were enrolled at different ages. More families declined to participate at 6 and 12 months, often reporting that they could not stay beyond their clinic appointment for these lengthier study visits when parenting and developmental outcomes collected as part of the main study took substantially longer (up to 60 minutes) than the 2-month enrollment visit (less than 10 minutes) when no outcomes were collected. There were no significant differences between cohorts for sociodemographic and cumulative risk comparisons, although this may have been harder to detect with our small sample size. Additionally, there are other factors that may be worth exploring as confounding variables, such as siblings’ age, maternal employment status, and the daily caregiving situation. Despite these limitations, our positive pilot findings are encouraging and indicate a need to rigorously test the longitudinal effects of our exceedingly simple intervention on child development, especially language and social-emotional skills.

## Conclusion

Our finger puppet intervention may provide an exceptionally simple, low-cost, and scalable way to encourage responsive caregiver-infant interactions promoting future language and social-emotional development. We found that puppets distributed at 2 months were associated with improved social-emotional developmental trajectories between 2 to 36 months of age when compared to families receiving the puppet at 6 and 12 months, suggesting that the optimal timing of the intervention is in early infancy. Our primary care-based approach has the potential to be widely disseminated for population-level promotion of early relational health. Our trajectory analysis also demonstrates a useful and potentially cost-effective approach to evaluating long-term developmental outcomes of an early intervention.

## Data Availability

The datasets used and analyzed during the current study are available from the corresponding author on reasonable request.

## Abbreviation

*ASQ-3*: Ages & Stages Questionnaires, Third Edition
